# Underperformance of Reverse-Transcriptase Polymerase Chain Reaction in Japan and Potential Implications From Diamond Princess Cruise Ship and Other Countries During the Ongoing COVID-19 Pandemic

**DOI:** 10.34172/ijhpm.2020.109

**Published:** 2020-06-28

**Authors:** Kana Yamamoto, Akihiko Ozaki, Yuki Senoo, Toyoaki Sawano, Tetsuya Tanimoto, Ranjit Sah, Jiwei Wang

**Affiliations:** ^1^Department of Reproductive, Developmental and Aging Sciences, Graduate School of Medicine, University of Tokyo, Tokyo, Japan.; ^2^Medical Governance Research Institute, Tokyo, Japan.; ^3^Department of Breast Surgery, Jyoban Hospital of Tokiwa Foundation, Fukushima, Japan.; ^4^Department of Surgery, Sendai City Medical Center, Miyagi, Japan.; ^5^Tribhuvan University Institute of Medicine, Kathmandu, Nepal.; ^6^School of Public Health, Fudan University, Shanghai, China.

## Dear Editor,

The coronavirus disease 2019 (COVID-19) emerged in Wuhan, Hubei province, China, in November 2019, and spread to multiple countries, leading the World Health Organization (WHO) to declare it a pandemic on March 11, 2020. As of June 20, 2020, 8 766 887 cases and 462 706 deaths had been reported, with a case fatality rate (CFR) of 5.3% worldwide.^1^ While a crude CFR of COVID-19 in Japan was reportedly similar at 5.3% on June 20, 2020,^1^ the CFR reported from different regions of the world vary widely, partly due to differences in the healthcare capacity and thoroughness of the crisis response and extent and progress of the epidemic.^2^ Administration of real-time reverse transcription polymerase chain reaction (RT-PCR) testing for potential COVID-19 patients and their surveillance and tracking are considered as the key response.^1^

In this regard, Japan’s situation is unique considering the initial intense opposition against the broad implementation of RT-PCR testing among authorities, primarily because of the reliability of RT-PCR testing. As a plausible explanation, there had been persistent concerns that the Japanese government may have intentionally limited the number of RT-PCR testing in Japan; the government initially limited testing to severe cases, returnees from abroad, and those who had come in close contact with the infected, while avoiding testing the remaining population, including asymptomatic individuals and those with mild conditions. Therefore, despite increasing recognition that an aggressive RT-PCR testing strategy is advisable with some limitations in March, it was only after Japan officially announced the postponement of Tokyo 2020 Olympic Games that RT-PCR testing increased in the country, although a causal relationship underlying this is unclear. At present, eligibility for RT-PCR is still determined by non-physician staff at local health centers after consultation from attending physicians. As a result, only 17 740 COVID-19 cases were reported as of June 20, 2020 in Japan,^1^ which is a relatively small number considering its population of 125.9 million people.^3^ Further, the number of RT-PCR testing in Japan (2.63/1000 persons) remained considerably lower than the average number of PCR testing in the Organisation for Economic Co-operation and Development (OECD) countries (60.56/1000 persons) from June 5 to 13 (data from France and South Korea are not available).^4^

To discuss the appropriate number of RT-PCR testing, broader contexts of this topic should be recognized. For example, the case of quarantine imposed on the cruise ship “Diamond Princess,” off the coast of Japan for 14 days (from February 5 to 19, 2020), could provide important information regarding the ongoing COVID-19 pandemic,^5,6^ considering that a large proportion of the 3711 cruise ship passengers and crew members underwent RT-PCR testing for COVID-19 (3011 respiratory specimens, with the exact number of people who underwent the test unknown), and those infected were rigorously tracked.^7,8^ Notably, approximately half of the passengers and crew members who tested positive for COVID-19 in the Diamond Princess were asymptomatic. According to the latest Japanese government report, there were 712 RT-PCR-confirmed cases (19.2% of the 3711 persons onboard) by April 30, 2020, of whom only 381 (53.5%) were symptomatic, while 331 (46.4%) were asymptomatic.^7^

This finding was similar to the finding reported in a recent article that reviewed the available evidence on asymptomatic severe acute respiratory syndrome coronavirus 2 (SARS-CoV-2) infection, in which asymptomatic persons presumably accounted for approximately 40% to 45% of SARS-CoV-2 infections,^9^ suggesting that the virus can spread silently and widely through human populations. These findings are in line with the current notion that a considerable proportion of COVID-19 patients remain asymptomatic before developing typical symptoms following the natural course of the infection. Therefore, an isolation strategy only for laboratory-confirmed cases with mild to severe symptoms is inadequate for containment. To ensure the security of high-risk populations (the elderly and those with comorbidities),^10^ it is imperative to thoroughly enact countermeasures, such as social distancing, quarantine, and isolation, against the spread of COVID-19, including among asymptomatic and pre-symptomatic individuals.^11^

Further, among the passengers and crew members with a confirmed infection, at least 13 deaths (1.8%) were recorded.^8^ Among the deceased passengers, five (38.5%) and four (30.8%) were reportedly in their 70s and 80s, respectively (the ages of the remaining three [23.1%] are unknown), according to the Ministry of Health, Labour and Welfare. Crude CFR of the passengers and crew members aboard the Diamond Princess was similar to that of Asian countries, such as South Korea, Thailand, and Malaysia. In South Korea, which set up an extensive testing program for COVID-19, the updated CFR was reported to be 2.3% (280/12 373, as of June 20, 2020).^1^ Likewise, CFRs of COVID-19 in Malaysia and Thailand were reported to be 1.4% and 1.8% (as of June 20, 2020), respectively.^1^

As such, we should recognize that the CFR on the cruise ship with a sufficient number of RT-PCR testing was much lower than that across Japan (5.3%).^12^ On Diamond Princess, all passengers were subjected to RT-PCR testing 6 days after the start of quarantine. By contrast, as shown in the OECD survey results, RT-PCR testing had not been fully implemented in Japan as of May 2020, and there could be many undiagnosed cases.^13^ The hypothesis can be underpinned by an estimate of excess death, which is often used to evaluate whether influenza and pneumonia deaths have increased as a result of a flu epidemic or not.^14^ Surprisingly, Japan has seen an increase in deaths (1056 persons) from March 1 to April 1, 2020 despite the absence of an influenza epidemic this season, suggesting that it was likely because of undiagnosed COVID-19 cases.^15-17^

It is noteworthy that two novel laboratory examinations (antigen testing using serum sample and RT-PCR testing using saliva sample) were officially introduced in June as alternative methods to RT-PCR using nasopharynx sample in Japan. Still, it is useless unless those novel methods are widely applied to contain the pandemic. To effectively prevent COVID-19 spread, performing more RT-PCR testing or other testing for potentially infected people is crucial, and we call for further discussions in academic fields on this matter. Therefore, despite decreasing dangers of the COVID-19 pandemic in Japan, the relevant stakeholders in the country should be aware of the lessons learned from Diamond Princess and other countries.

## Acknowledgments

The authors thank Dr. Masahiro Kami for his constructive opinions and insights.

## Ethical issues

Not applicable.

## Competing interests

AO and TT report personal fees from Medical Network Systems (MNES) Inc., outside of the work. The other authors declare no conflict of interests.

## Authors’ contributions

KY, YS, and AO acquired, had full access to and control of all data, and oversaw all data analyses. All authors were involved in the study concept and design. All authors were involved in the analysis, interpretation of results, and formation of conclusions.

## Authors’ affiliations

^1^Department of Reproductive, Developmental and Aging Sciences, Graduate School of Medicine, University of Tokyo, Tokyo, Japan. ^2^Medical Governance Research Institute, Tokyo, Japan. ^3^Department of Breast Surgery, Jyoban Hospital of Tokiwa Foundation, Fukushima, Japan. ^4^Department of Surgery, Sendai City Medical Center, Miyagi, Japan. ^5^Tribhuvan University Institute of Medicine, Kathmandu, Nepal. ^6^School of Public Health, Fudan University, Shanghai, China.

## References

[1] Worldometer. COVID-19 coronavirus pandemic. https://www.worldometers.info/coronavirus/#countries. Updated June 20, 2020. Accessed June 20, 2020.

[2] Onder G, Rezza G, Brusaferro S (2020). Case-fatality rate and characteristics of patients dying in relation to COVID-19 in Italy. JAMA.

[3] Statistics Bureau of Japan. Preliminary counts of population of Japan. https://www.stat.go.jp/english/index.html. Updated May 1, 2020. Accessed June 17, 2020.

[4] Our world in data. Coronavirus (COVID-19) testing statistics and research (June 13, 2020 update). https://ourworldindata.org/coronavirus-testing. Updated June 13, 2020. Accessed June 13, 2020.

[5] Sawano T, Ozaki A, Rodriguez-Morales AJ, Tanimoto T, Sah R (2020). Limiting spread of COVID-19 from cruise ships - lessons to be learnt from Japan. QJM.

[6] Mizumoto K, Kagaya K, Zarebski A, Chowell G (2020). Estimating the asymptomatic proportion of coronavirus disease 2019 (COVID-19) cases on board the Diamond Princess cruise ship, Yokohama, Japan, 2020. Euro Surveill.

[7] National Institute of Infectious Diseases. Field briefing: Diamond Princess COVID-19 cases, 20 Feb Update. https://www.niid.go.jp/niid/en/2019-ncov-e/9417-covid-dp-fe-02.html. Updated February 21, 2020. Accessed June 20, 2020.

[8] Situation update for COVID-19 and the MHLW’s response (May 1, 2020 updated). https://www.mhlw.go.jp/stf/newpage_11146.html. Updated May 1, 2020. Accessed May 3, 2020.

[9] Oran DP, Topol EJ (2020). Prevalence of asymptomatic SARS-CoV-2 infection: A narrative review. Ann Intern Med.

[10] Wu C, Chen X, Cai Y, et al. Risk factors associated with acute respiratory distress syndrome and death in patients with coronavirus disease 2019 pneumonia in Wuhan, China. JAMA Intern Med 2020;E1-E10. doi:10.1001/jamainternmed.2020.0994. PMC7070509 32167524

[11] Anderson RM, Heesterbeek H, Klinkenberg D, Hollingsworth TD (2020). How will country-based mitigation measures influence the course of the COVID-19 epidemic?. Lancet.

[12] World Health Organization. Coronavirus disease 2019 (COVID-19) situation report-105. https://www.who.int/docs/default-source/coronaviruse/situation-reports/20200504-covid-19-sitrep-105.pdf?sfvrsn=4cdda8af_2. Updated May 4, 2020. Accessed June 20, 2020.

[13] Organisation for Economic Co-operation and Development. Testing for COVID-19: A way to lift confinement restrictions. http://www.oecd.org/coronavirus/policy-responses/testing-for-covid-19-a-way-to-lift-confinement-restrictions-89756248/. Updated May 4, 2020. Accessed June 14, 2020.

[14] Our world in data. Excess mortality from the Coronavirus pandemic (COVID-19). https://ourworldindata.org/excess-mortality-covid. Updated June 19, 2020. Accessed June 20, 2020.

[15] Sakamoto H, Ishikane M, Ueda P (2020). Seasonal influenza activity during the SARS-CoV-2 outbreak in Japan. JAMA.

[16] National Institute of Infectious Diseases. Report of influenza and pneumonia deaths in 21 major metropolitan areas for the 2019/20 season by the rapid identification system for influenza-related deaths. https://www.niid.go.jp/niid/ja/flu-m/2112-idsc/jinsoku/1852-flu-jinsoku-7.html. Updated May 24, 2020. Accessed June 20, 2020.

[17] Statistics of Tokyo. STATISTICAL DATA. https://www.toukei.metro.tokyo.lg.jp/homepage/ENGLISH.htm. Updated December 25, 2020. Accessed June 17, 2020.

